# Malaria Control Programme in Nigeria: uptake of prevention strategies - a systematic review

**DOI:** 10.4314/ahs.v24i2.21

**Published:** 2024-06

**Authors:** Collins Ugwu, Ngozi Ugwu, Ogbonnaya Ogbu, Onyedikachi Chukwu, Nneka Chika-Igwenyi, Olaronke Afolabi, Daniel Igwe, Esther Ekwe, Ngozi Ezekwesili, Chigozie Uneke, Godsent Isiguzo

**Affiliations:** 1 Ebonyi State University, Faculty of Clinical Medicine, Internal Medicine; 2 Federal University Teaching Hospital, Abakaliki, Hematology; 3 Ebonyi State University, Faculty of Biological Sciences, Applied Biology; 4 Chemical Pathology, Alex Ekwueme Federal University Teaching Hospital Abakaliki, Nigeria; 5 Department of Internal Medicine, Alex Ekwueme Federal University Teaching Hospital Abakaliki; 6 Alex Ekwueme Federal University Teaching Hospital, Abakaliki, Medical Microbiology; 7 University of Nigeria Teaching Hospital, Internal Medicine; 8 Ebonyi State University, Medical Microbiology, Faculty of Medical Sciences; 9 Ebonyi State University, African Institute for Health Policy & Health Systems

**Keywords:** Anaemia, malaria control strategies, Nigeria, Uptake

## Abstract

**Background:**

Malaria presents a colossal burden to Africa, including Nigeria. The objective of this study was to review relevant publications to identify specific malaria control strategies in Nigeria and to determine their level of uptake.

**Methods:**

A Medline Entrez Pubmed search was conducted to identify studies from July 2013 to June 2018 investigating malaria control strategies. The search yielded 123 publications and twelve publications that met the inclusion criteria were systematically reviewed and results presented.

**Results:**

Five publications investigated the level of uptake of IPTp-SP and all reported low uptake of IPTp-SP. Five other publications investigated the uptake of LLINs, of which two reported good uptake. Two studies were on the uptake of mRDT or microscopy before Artemesinin-based combination therapy (ACT) and reported good uptake. Factors associated with poor uptake of malaria preventive strategies included a poorly-financed and poorly structured healthcare system, poor antenatal clinic visits, unavailability of the antimalaria drugs and nets, ignorance, poverty, cultural/religious belief and cost of mRDT and microscopy.

**Conclusion:**

Though malaria control strategies are available in Nigeria, there was insufficient uptake of these preventive strategies. Awareness creation and education on the importance of preventive strategies and their efficient utilization will help reduce Nigeria's malaria burden.

## Introduction

Malaria is a common tropical disease with significant economic, social, and morbidity consequences^1^. The number of nations and territories where malaria is still an endemic problem is down from 108 in 2000 to 91 as reported by WHO in 2014. More than half of the world's population continues to be at risk for malaria, with sub-Saharan Africa continuing to have the greatest frequency worldwide and having 43 nations that are endemic for the disease^2^. Africa is heavily burdened by malaria, which also continues to impede the continent's economic growth. Approximately 60% of all outpatient visits and 30% of all hospital admissions in Nigeria are due to this disease, which continues to be one of the nation's most critical public health issues^3^. Nearly 110 million clinical cases and 300,000 annual fatalities, including up to 11% of maternal mortality, are attributed to it. Additionally, malaria has a significant negative economic impact, costing around N132 billion ($172,597,656.00) yearly in treatment expenses, lost productivity, and other factors^2^. In Nigeria, as in many other endemic countries in Africa, pregnant women and young children suffer the most from malaria. It was thought that 3-5% of maternal anaemia, 8-14% of low birth weight (LBW), and 3-8% of infant mortality were caused by malaria during pregnancy^4^. Previous studies in south-west Nigeria found that between 60 and 72% of pregnant women had malaria parasites^5^,^6^.

The parasite of the genus Plasmodium that causes malaria is often spread through the bite of an infected female anopheles mosquito. The most prevalent species of malaria parasite in Africa, *Plasmodium falciparum*, causes up to 98% of cases in Nigeria^3^. *P. malariae, P. ovale*, and *P. vivax* are examples of additional species. The majority of Nigeria is plagued by malaria, which has a steady annual transmission rate. One or more of the variables that promote mosquito breeding and malaria transmission are high temperatures, high humidity, and rain^3^. Geographical location, epidemiology, immunity and age, all affect how severe the clinical signs and symptoms of malaria are. Investigating host immune response and parasite invasion mechanisms is necessary to comprehend the intricate pathophysiology of malaria. Malaria sympoms include a headache, nausea, vomiting, chills, and regular cycles of fever. Cerebral malaria, pulmonary oedema, acute renal injury, hypoglycaemia, lactic acidosis, anaemia, and liver involvement are some of the severe signs that frequently show clinically^7^. People who have never before been exposed to the disease, such as young children and tourists from non-endemic regions, frequently develop severe malaria. One of the major problems to global public health in the twenty-first century is the management of vector-borne illnesses like malaria. Numerous studies have demonstrated the effectiveness of malaria prevention strategies, yet most settings still lag behind in implementing them. The creation of vector control tactics has received a lot of attention in the fight against malaria. High-risk groups including expectant mothers and young children are the focus of additional protective measures.

Vector control is one method of preventing malaria, and it comprises taking actions against disease vectors in order to reduce their capacity to spread the illness^8^. The use of long-lasting insecticidal nets (LLINs), where the pesticide lasts for up to 1-3 years, is one of the primary vector control strategies. Other vector control techniques include indoor residual spraying (IRS) and larval life-cycle management, which deals with controlling aquatic areas that could serve as mosquito breeding grounds in order to prevent the embryonic growth of the insects.

By providing vulnerable groups in malaria endemic nations with malaria protection, the morbidity and mortality linked to malaria can be decreased. Pregnant women, children under the age of five, nomadic people, internally displaced persons as a result of terrorists and banditry activities, herdsmen and farmers clashes in Nigeria and travellers to countries with endemic diseases are among the susceptible populations. In malaria-endemic regions of Africa, where 25–30 million pregnant women are at risk of *P. falciparum* infection and associated negative effects and problems during pregnancy, pregnancy and malaria continue to be major public health concerns4. Intermittent preventive therapy with sulphadoxine-pyrimethamine (IPTp-SP) is used as preventative therapy in pregnant women in the majority of sub-Saharan African nations after the first trimester^9^. It has been demonstrated that this chemoprevention lowers maternal mortality as well as maternal anaemia, low birth weight, and perinatal death.

The integrated vector management (IVM) strategy, prompt diagnosis and appropriate treatment of clinical cases at all levels and in all sectors of health care, and prevention and treatment of malaria in pregnancy were the three main interventions included in the Nigerian malaria control program's establishment of a reliable system for malaria control in 2008^10^. A National Malaria Strategic Plan was put into place by the malaria control program in 2014 with the goal of achieving pre-elimination status (less than 5000 cases per 100,000 people) and eliminating all malaria-related deaths by 2020^10^.

According to the World Health Organization and the national malaria control program, the uptake of malaria prevention and management strategies is inadequate, particularly in Nigeria^11^,^12^. The purpose of this study was to analyse pertinent literature in order to identify malaria prevention and control strategies in Nigeria, and factors that limit the uptake of malaria prevention intervention strategies.

## Materials and methods

This review started with a careful search for the publications on studies that investigated malaria control strategies in Nigeria. In order to identify relevant publications with high degree evidence, the search was restricted to PubMed database. This was due to the global recognition given to PubMed database for having publications with high quality. Journals indexed in the database have been noted to be mainly peer-reviewed journals. Therefore, a MEDLINE entrez PubMed search was conducted and studies done from July 2013 to June 2018 were screened. Studies that were published in English language, which investigated malaria control programmes- the uptake or utilization of the control strategies in Nigeria, were sought for. The following inclusion criteria for the study were applied: research done in Nigeria, primary scientific investigations, not reviews, Studies that targeted Nigerian program to combat malaria, Studies that evaluated the uptake of the malaria control strategies, Studies which have policy-relevant evidence and implementation studies.

The following search terms / key words were used in the search: “malaria control programme, intermittent malaria treatment in pregnancy, malaria rapid diagnostic test, Nigeria”. Three people did the screening of the articles and when there were contradictions, they all met and agreed on the articles to include based on inclusion and exclusion criteria for the study. The search yielded 123 publications which were grouped into 3 categories depending on the type of malaria control measure they addressed. Adoption of a pregnancy-specific intermittent preventive treatment (IPTp) in Nigeria yielded forty-two (42) publications (Category 1), Uptake of long lasting insecticide-treated net (LLIN) in Nigeria yielded fifty-one (51) publications (Category 2), uptake of malaria rapid diagnostic test (mRDT) and microscopy for diagnosis of malaria yielded thirty (30) publications (Category 3) making a total of 123 publications.

**Figure 1:**
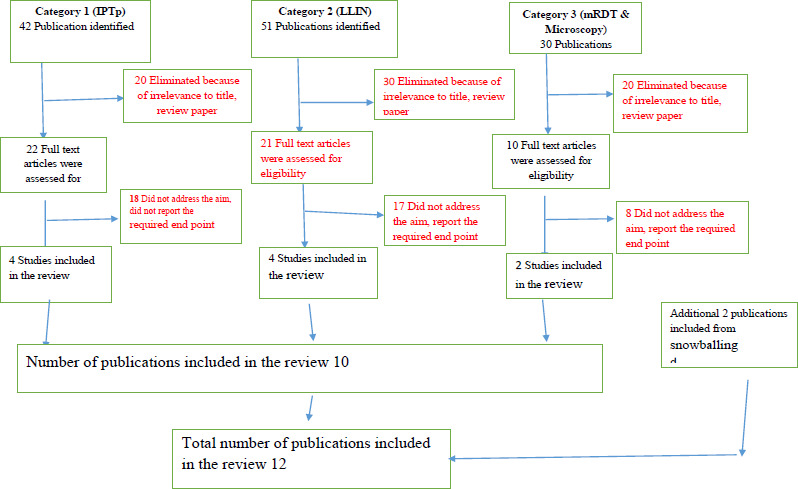
Flow Chart of Publication Search Protocols

The inclusion criteria for the study were applied to the one hundred and twenty-three (123) publications in order to determine which ones addressed the relevant problem and accomplished the study's goals. The publication selection process took a three-stage process. This was done first, by taking a critical look at their “Titles”. Those that are not related to the subject matter, or not carried out in Nigeria or that are not primary studies did not receive further attention and were excluded. The second stage followed, and involved taking a critical look at their “Abstracts”. During this stage, some publications that did not meet the study criteria were further excluded. Hence, where the “Abstract” appeared relevant, the full publication was sought for inclusion into the review. Ten papers were identified to have satisfied the study inclusion requirements and were chosen after these procedures. Two additional publications were also added from snowballing and review of the references of the selected publications, making a total of twelve publications selected for the review (Figure 1).

### Data Analyses

The publications were subjected to content analysis and critical review. The following information were considered relevant: the author(s), the year of publication, the state in which the implementation of the control program occurred, the kind of implementation strategies, the study's design, sampling procedure, sample size, season of the year, the population it was intended to reach or its data source, the evidence it produced, and the study's policy implications, including its advice. The information was sought and presented.

## Results

The twelve studies identified to have met the study inclusion criteria were used for this review and the outcomes presented using tables. For the purpose of ease of presentation and discussion, the publications were categorized into three according to the malaria control strategies addressed in the programme or project (Tables 1- IPTp, Table 2- LLIN and Table 3- mRDT & Microscopy).

**Table 1:** Profile and characteristics of scientific publications associated with intermittent preventive treatment in pregnancy (IPTp) with Sulfadoxine-Pyrimethamine in Nigeria

Author/year of publication	State	Health issue in review	Scope/nature of control strategy	Study design	Sampling technique	Sample size	Seasons of the year	Target population	Evidence generated/outcome	Policy implication/Recommendation
Yaya et al., 2018	Nation wide survey	Uptake of IPTp-SP	Malaria indicator survey	Retrospective	Key informant interview	8,111	Not stated	Pregnant women	Low uptake of IPTp-SP	Strengthen policy on IPTp
Olukoya et al., 2017	Nationwide survey	Missed opportunity of IPTp-SP	Health survey on uptake of IPTp-SP	Cross-sectional	Stratified sampling technique	6,910	2 seasons	Women 15-49 years (preg within the last 2yrs	Very high missed opportunity for IPTp-SP	Stronger government commitment to enhance IPTp-SP uptake
Mohammad et al., 2016.	Yobe	Uptake of IPTp-SP	Malaria prevention and delivery outcome.	Cross-Sectional	Consecutive Interviewer administered questionnaire	184	Rainy season	Pregnant women at FMC Nguru	Poor uptake of IPTp-SP.	To emphasize malaria preventive strategy among displaced pregnant women
Esu et al., 2013	Cross River.	Uptake of IPTp-SP	Utilization of IPTp in Cross River	Retrospective	Simple random sampling	322	Dry season	Pregnant women in 36 health facilities	Poor utilization of IPTp-SP in Cross River State	Efforts to ensure early antenatal booking and uptake of IPTp.
Onyebuchi et al., 2014	Ebonyi	Uptake of IPTp-SP	Adherence and acceptance of IPTp-SP	Prospective descriptive study	Purposive and consecutive enrolment	516	2 seasons	Pregnant women in two Obstetric centres	Poor adherence to IPTp-SP	Improve access and acceptability of IPTp-SP

**Table 2:** Profile and characteristics of scientific publications associated with uptake of long lasting insecticide treated net (LLINs) in Nigeria

Author/year	State	Health issue in review	Scope/nature of control strategy	Study design	Sampling Technique	Season of the year	Sample size	Target population	Evidence generated/outcome	Policy implication/recommendation
Ejembi et al., 2018	FCT Abuja	ITNs uptake	ITNs use among displaced people	Cross Sectional	Cluster sampling	Not stated	393	Under-5 children in IDP camp	High net utilization	ITNs distribution and hanging campaign.
Israel et al., 2018	Osun	ITNs uptake	ITNs use among under 5	Cross Sectional	Multistage sampling	Not stated	1020	Mothers/care givers of under-5	Good utilization of ITNs among under 5	Intensify health education to improve utilization.
Esimai and Aluko, 2014	Osun	ITNs uptake	ITNs use among under 5	Cross Sectional	Multistage sampling	Not stated	200	Care givers of under-5	Poor utilization of ITNs	Intensive awareness creation about ITNs
Ankoma et al., 2014	21 States survey	ITNs uptake	ITNs use in Pregnant women	Cross Sectional	Multistage sampling	Not stated	2,348	Pregnant women	Poor uptake of ITN	Enhance communication to better uptake
Osuorah et al., 2013	Nation wide survey	ITNs uptake	Household use of ITNs	Cross Sectional	Multistage sampling	Not stated	5,895 households	Under-5	Poor ownership and uptake	Effort towards ownership and uptake

### Uptake of intermittent preventive therapy among Pregnant women in Nigeria

Yaya et al., evaluated the prevalence of IPTp uptake and the patterns of IPTp uptake among different educational and wealth categories adjusted for relevant socio-demographic factors^13^. Basic demographic and socioeconomic information of the sample population showed that 18,603 women aged between 15–49 years were included in the study. Mean age of the participants was 28.76 years (SD 7.01). The overall prevalence of taking adequate doses of IPTp-SP during the latest pregnancy was 29.5%. The study showed negative association between the uptake of IPTp-SP with educational status, and positive association with wealth status.

Olukoya et al., evaluated missed opportunity for IPTp-SP and attempted to look at correlates and predictors^14^. The study found the rates of missed opportunity (using two doses as standard for determining missed opportunity for IPTp-SP) as 73.4% giving an uptake of two doses at 26.6%. If 3 doses were used as standard for determining missed opportunity for IPTp-SP, miss opportunity increased to 88.6% and uptake reduced further to 11.4%. The highest rates of missed opportunity for IPTp-SP were reported among the more educated women (78.5%) while the lowest rates (61.6%) were reported among women with no education (p<0.001). Missed opportunity was higher among the wealthier ones with 80.2% compared to 68.9% among women in the poorer wealth quintile (p<0.001). Rates of missed opportunity for IPTp-SP were higher among respondents in urban (76.0%) than rural (69.6%) areas (p=0.001). Approximately 77.0% of women with timing of their first ANC in the third trimester reported missed opportunity for IPTp-SP compared to 72.1% of women whose first ANC was in the second trimester (p=0.038).

In Yobe State, Muhammad et al., studied 184 women and found that only 80 (43.0%) of the parturient women received up to two or more doses of IPTp-SP, while 57.0% received only a dose or none. Majority of the women who had taken up to two doses were primi- and secundi-gravidae 45 (40.0 %), while only 35.0 % of the multi-gravidae had taken up to two doses^15^.

In Cross River State, Esu et al., studied the record of 322 pregnant women who registered for ANC in 36 facilities^16^. They assessed their antenatal visits and uptake of IPTp-SP and the gestational age at booking. The result showed low uptake of IPTp-SP with the percentage of women who received two or more doses of SP was 99 (30.7%). In terms of gestational age (GA) at booking, the uptake was more with those who booked at second trimester (32.3%) when compared to those who booked at the third trimester (19.1%). The relationship between IPTp received and GA at booking was statistically significant (χ^2^ = 12.81; degrees of freedomdf = 6; P = 0.0462). There was also significant difference in the doses of IPTp received between women that had the first dose in the second trimester when compared to those that had it in the third trimester (χ^2^ = 7.124; df = 2; P = 0.0284).

In Ebonyi State, Onyebuchi et al., studied 516 women and reported that the uptake of just a dose of IPTp-SP was 72.1% (367/516)^17^. The adherence to second doses of IPTp was 59.7% (219/367), and only 4.9% (18/367) took a third dose. The reasons for poor adherence after the first dose were “not having money to pay for drugs” (31.5%), “felt that there was no need for the drug” (l6.9%), “forgot to take the drugs” (12.3%) and “first dose caused weakness” (7.7%). Clinical malaria was seen in 85% (127/149) of women who did not receive IPTp at all compared to 20.5% of those who received at least one dose of IPTp (RR 2.53; 95% CI 2., 3.04; p<0.001). All those who had clinical malaria despite IPTp had only one dose of IPTp. Malaria in pregnancy was significantly more in women who did not adhere to subsequent doses of IPTp than in those who adhered (24.6% versus 14.3%, respectively; risk ratio =2.5; 95% CI; 2.1, 3.0; P=0.001). Neonatal malaria occurred significantly in neonates whose mothers did not receive IPTp compared to those whose mothers received at least one dose of IPTp (7.4% versus 3.4%; risk ratio =1.4; 95% CI 0.9, 2.1; P=0.003). Women who did not receive IPTp at all delivered lower birth weight babies compared to neonates born to women who took at least one dose of IPTp (10.5% versus 3.7%; RR =2.84; 95% CI 1.4, 7.2; P = 0.001) (Table 1).

### Uptake of long lasting insecticide treated nets

In a camp for internally displaced people in Abuja, Ejembi et al. evaluated the factors that predicted whether or not children aged 5 to 59 months would use insecticidal treated nets. The 30 kids who did not sleep under the LLIN the night before the poll gave a variety of explanations, including “the net being too hot” 7 (25.0%), “not enjoying the fragrance” 7 (25.0%), and “net not haung” 5(16.7 percent). The “room was too tiny,” “yet to wash the LLIN” as advised, and had employed “other mosquito prevention measures” 11(33.3%) were further justifications.18. Even though just 26 households had the optimal person-net ratio, the survey found that 182 (75.6%) households owned at least one LLIN, resulting in a coverage rate of 11.2 percent. Additionally, they discovered that 260 of the 290 children aged 6-59 months living in LLIN-owning households used one the night before the study, resulting in an LLIN use rate of 89.7%.

Israel and his team in Osun State assessed 1,020 mothers' and caregivers' awareness of and use of LLIN among under-5-year-old children in six specific areas^19^. The study discovered that 836 (82.9%) of the respondents possessed at least one LLIN, and 591 (58.6%) of them said their children under the age of five had slept beneath the net the night before the poll. Some under-five children do not sleep under LLIN for a variety of reasons, including “excessive heat,” cited by caregivers 402 (96.4%), “reactions to chemicals,” cited by caregivers 315 (75.5%), “unpleasant odour,” cited by caregivers 172 (41.3%), “cost is expensive,” cited by caregivers 25 (6%) and “no mosquitoes in the house,” cited by caregivers 48. (11.5%). Formal education of caregivers (aOR 1.4, 95% CI 1.0-2.1) and their familiarity with LLIN were the factors that determined LLIN use (aOR 1.8, 95% CI 1.4–2.5).

Esimai and Aluko evaluated the usage of insecticide-treated nets and the factors influencing its use among those who care for children under the age of five in a different study conducted in Osun State^20^. Only 34.4% of the respondents, of which 30.3% received it for free, had access to LLIN, according to their survey. A third (32.8%) had never used the net, and 18.5 percent of people currently used LLIN, of which 32.8% were owners. “Unavailability” (13.0%) and “expensive” were two of the explanations cited by 67.2% of those who had never used the net (13.7%). The respondents listed a few difficulties with ITN use, including “not easy to treat” (0.1%), “difficult to set up” (0.1%), and “no place to maintain it” (0.2%).

In a survey of 2,348 pregnant women in 21 states of Nigeria, Ankoma et al. found low uptake and that exposure to mass media was positively associated with sleeping under an ITN, with those who listened to radio having an approximately two-fold higher tendency to do so (OR = 1.56; 95% CI 1.07 to 2.28; P = 0.02) and those who had heard of the campaign having an OR of 1.53 (OR = 1.53; 95% CI 1.07 to 2.17;

Only 2,489 (48.5%) of the 5,146 under-5 children assessed by Osuora et al., according to data from a national study, slept under a bed net^22^. Only 2,673 (45.5%) of the households in the survey had bed nets. When compared to urban households, whose net ownership was roughly 38% (37.9%), rural areas had a higher percentage (49.3%) (P = 0.001). The majority (52.0%) of poorer households owned bed nets. The next highest proportion of bed net ownership was found in middle-class homes (51.1%), while the lowest rate (38.6%) was found in wealthy households (P 0.05). Surprisingly, the percentage of households with a head with no formal education was the largest (51.7%), followed by those with only elementary education (42.9%) and those with greater educational attainment (40.6%) (P 0.05). The lack of mosquitoes in the room (19.7%), the room being too hot (27.7%), the difficulty of hanging the bed net (25.4%), dislike of the smell (2.8%), the feeling that the child is confined or restrained (3.8%), a problem with the net, such as being torn, old, or dirty (17.1%), an unsafe chemical being used to treat the net (2.8%), and the net inducing coughing (1.0%) were the factors that led to a child not sleeping under the net. (Table 2).

### Uptake of malaria rapid diagnostic test (mrdt) and microscopy

Nigeria started a phased systematic deployment of rapid diagnostic tests (RDT) at the level of primary health care (PHC) facilities in 2011, however despite numerous attempts, the country's national malaria testing rate is still incredibly low, and RDT uptake has been inconsistent.

In order to ascertain the provider and patient perspectives of RDT use at the PHC level and its implications for enhancing uptake and compliance, Mokuolu et al. conducted a survey in 120 randomly chosen PHCs in Nigeria. Ninety percent of the health professionals surveyed correctly identified the required quantity as a drop, 99.2 percent claimed to being able to perform an RDT, and 90.8% said they would utilize the test results to decide which patients should receive ACT. They cited the following justifications: they had greater confidence in the results than microscopy results (43.1%), it would limit the unnecessary use of ACT (87.2%), it would allow for the study of other conditions as a cause of fever (67.9%), and it would reduce the usage of ACT overall (Table 3).

Seventy-three percent of health professionals were asked about their compliance with RDT outcomes in terms of their procedures. In contrast, if the test came back negative, 32% of the healthcare professionals said they would prescribe ACT, 30% would give antibiotics, 31% would refer these individuals, and 7% would take no further action. Patients who underwent RDT were aware that it detected malaria (96.7%), believed it was necessary (94.9%), and welcomed the test (98.8%) because it increased their confidence in the diagnosis (92.3%).

In formal private health institutions in Nigeria, Mokuolu et al. evaluated the level of usage of malaria rapid diagnostic testing (RDT), compliance with test results, and associated problems^24^. Regarding malaria testing procedures, responses from 232 facilities' health workers revealed that 98 (42.2%) of those facilities used microscopy, 46 (19.8%) used RDT, 74 (31.8%) used both RDT and microscopy, and 16 (6.8%) said they did not perform any malaria tests at all. 107 (44.6%) of the 240 healthcare facilities had RDT on hand, though 110 (45.8%) regularly used it.

1,533 of the 2,077 patients who presented to the institutions with a febrile illness had blood tests performed while they were there, for a testing rate of 73.8% (95% CI 71.7–75.7 percent). The study found that out of 1,078 patients whose malaria test results were reported positive, 892 (82.7%) received ACT, while out of 181 patients whose malaria tests were reported as negative, 126 (69.6 percent) did not receive ACT. Based on compliance with test results, the study showed a compliance of 80.9 percent (Table 3).

## Discussion

This review included publications on the various malaria control strategies in Nigeria such as IPTp, LLIN, mRDT and microscopy and the uptake of the malaria control intervention strategies among different target groups. The outcome of this review showed that there was poor uptake of malaria control strategies generally which was attributed to some factors. These factors included poorly organized and underfunded healthcare system as well as cultural and socio-demographic barriers.

Uptake of intermittent preventive therapy among pregnant (IPTp-SP) women in Nigeria.

All the five publications showed poor uptake of intermittent preventive therapy among pregnant women in Nigeria. Sanni et al., in a nationwide malaria indicator survey in 2015 reported an uptake of 25% among pregnant women^25^. Olukoya et al., reported uptake of at least two doses of Sulfadoxine-Pyrimethamine at 26.6% representing missed opportunity for IPTp-SP at 73.4%^14^. Muhammad et al., in a study in Yobe State reported uptake of 43.0%^15^ while Esu et al., in Cross River State in a study among pregnant in 36 health facilities reported uptake of IPTp-SP at 30.7%^16^. Onyebuchi et al., equally reported poor uptake of IPTp-SP among pregnant women in Ebonyi state^17^. These results were comparable to those of a study carried out in Uganda by Sangare et al., where the uptake was 31%^26^ and Andrew et al., in a survey conducted in 31 sub-Saharan African countries reported a median uptake of 29.6%^27^. However, higher uptake of 43% was reported in Zambia and 60% in Ghana probably because of better organised healthcare system, though still far below the recommendation of WHO^25^.

The poorly organized and underfunded health care system in Nigeria may be to blame for the low uptake or higher rates of missed opportunity for IPTp-SP that were seen in the studies as well as cultural and socio-demographic barriers. This may be the cause of the inadequate integration of the national health care system's reproductive health division's malaria control program, which lacks the resources necessary to carry out the program effectively. The higher uptake in these countries could be attributed to improved healthcare system as well as the fact that they were among the earliest countries to approve IPTp policy^28^.

According to the findings of the recent studies, Nigeria has not been able to reach one of the Roll Back Malaria initiative's goals of having 100% of pregnant women receive intermittent preventative therapy for malaria by 2015^29^. These results indicate that more has to be done by the government to reverse the trend. A well-designed health system, adequate funding, and efforts to overcome the noted cultural and sociodemographic barriers to IPTp-SP uptake, as well as providing the necessary logistics to optimize IPTp-SP within the scope of ANC, could be a potential strategy.

Uptake of IPTp-SP was found to have negative association with level of education, urban dwellers and late booking at the antenatal clinic. This can be due to the fact that educated women were more likely to work longer hours and hence did not fully benefit from ANC visits. A cross-sectional study of rural Kenyan women found that those with formal education were more likely to have received at least one dose of IPTp-SP^30^. In Malawi and Uganda, it was discovered that there was a favourable correlation between educational attainment and IPTp-SP uptake^31^, ^26^. With regards to pregnancy characteristics, these studies had similarity with others which showed that parity and timing of first visit to ANC were determinants of missed opportunity as shown in Ghana^32^,^33^,^34^. Primips and Secundi-para were reported to have better uptake than multi-para. This could be due to over confidence on the part of the multi-para who were more experienced than the primips and secundi-paras. Presentation/booking at first or second trimester were found to have better uptake than those who booked at third trimester. This is not surprising considering the fact that those who booked at first or second trimester had more time ahead of them to have taken two or more doses since the drug is usually given one month apart. There were also conflicting results on the association with the level of uptake and social status. While some reported positive association with higher uptake among the wealthy respondent, other studies stated otherwise.

### Uptake of long lasting insecticide-treated nets

Five publication investigated the uptake of long lasting insecticide-treated net (LLIN). Two out of these five studies reported good uptake^18^,^19^ while three publications reported poor uptake of insecticide treated nets^20^, ^21^, ^22^.

The Study by Ejembi et al., among children age 5 to 59 months in the internally displaced persons (IDP) camp in FCT Abuja showed high net ownership and also utilization (89.7% uptake), while the coverage was low^18^. LLIN seen hung over sleeping areas and the type of camp site were the review's independent predictors of use. According to the study, households with IDPs between the ages of 6 and 59 months had a high ownership rate of LLIN (76.7%). The ratio is, however, less than what the WHO advises^2^. However, the ownership rate in the research was greater than the LLIN ownership rate nationally and in the Federal Capital Territory Abuja, which was 69 percent and 42 percent, respectively, among infants younger than 59 months^35^. The uptake of 89.7% at the IDP camp in Abuja was higher than 58.6% as reported in Ogun State by Israel et al.,^19^, 18.5% uptake by Esimai and Aluko,^20^ and 48.5% uptake as reported Osuora et al.,^22^.

The fact that many LLINs at the IDP camps were distributed through NGOs, the government malaria elimination program, and religious organizations rather than being purchased may be the cause of the high ownership of LLIN there. Despite the fact that ownership does not correlate utilization^36^, it was nevertheless a positive step toward meeting the world's goals for the eradication of malaria. The high rate of ownership discovered in this study was comparable to that discovered among IDPs in Uganda, where the rate was 75.6%^37^. The high ownership of LLIN recorded in these studies could be due to the fact that the nets were given out free of charge and the respondents did not pay for them. The results were consistent with reports from earlier research conducted in malaria-endemic nations like Kenya (52.2%) and Burkina Faso (70.0%). A Ghanaian study reported a reduced usage rate (43.0 percent) among children under the age of five, which was linked to the respondents' perception that mosquitoes were scarce or non-existent in their environment and that malaria was a benign health issue^40^.

Only 16.1% of children under the age of five slept under an LLIN the night before the survey, according to a 2013 data from the Nigerian Demographic and Health Survey^10^. Nevertheless, the 2015 Nigeria Malaria Indicator study found improvements in the use of LLIN, with 43% of children under the age of five sleeping under one the night before the survey^35^. Although LLIN use appears to be gradually increasing, the objective was to reach at least 80% in order to decrease malaria morbidity and mortality^41^. This was in contrast to the findings from the Democratic Republic of the Congo, where just 34% of homes in IDP camps had LLIN, the net service was not entirely free, and most homes could not afford to buy the net^36^.

Israel et al., reported that higher educational status of the mother had positive effect on net uptake^19^. Arogundade et al., and Ifezulike et al., also reported positive influence of higher educational status of the mothers on the uptake LLIN in children^42^,^43^. This is due to the fact that education will improve knowledge and will help to dispel misconceptions with proper attitudinal change and good behaviour. Children under the age of five frequently report extreme heat, chemical reactions, disagreeable odours, and cost as barriers to using LLIN. These results were consistent with the usage barriers discovered in studies carried out in Ghana and Kenya, where it was discovered that knowledge was positively linked with LLIN use by children under the age of five^44^,^38^.

### Uptake of rapid diagnostic test and or microscopy for diagnosis of malaria

Out of the two studies on the uptake of rapid diagnostic test or microscopy for malaria, one centred on public primary health centre and the second study was among formal private facilities^23^,^24^. The uptake of RDT was good at both PHC and private health facilities. The compliance rate was also high. This is in line with data from other nations where studies in Sudan and Uganda^45^,^46^ and elsewhere have revealed substantial RDT penetration among health personnel at the PHC levels. About one-third would prescribe antibiotics for RDT negative results, one-third would refer patients, and one-third would prescribe ACT. Similar reports from Tanzania^47^ can be found here. As was described by Baltzell et al. in Zanzibar, Tanzania^48^, having an integrated quick diagnostic test kit that would simultaneously test for malaria, bacterial infections, and possibly viral pathologies may be the direction to explore.

This review's sub-group analysis revealed that health professionals' decisions to recommend ACT to RDT negative subjects were significantly influenced by the parents' educational attainment. The governments' noble desire to make ACT medications freely available may be compromised by these major issues affecting ACT use in RDT negative people. This indicates that it is impossible to ignore the impact of the general population on health professionals' adherence to RDT outcomes. As a result, effective information, education, and communication/behavioural change communication (IEC/BCC) initiatives that would specifically target community members are required.

When comparing the results of the RDT testing to the clinical picture, there may have been probable scepticism on the part of the healthcare professionals. If there is any uncertainty regarding the results of the RDT testing, a second test should be conducted. This second test may consist of an RDT with a different mechanism of action or a microscopic examination. Malaria should be ruled out as a possible cause of fever if both tests are negative^29^. Additional tactics to enhance the quality assurance system for RDTs include the use of positive control wells in the field to increase providers' trust in the results^49^. Health professionals should avoid treating malaria empirically and instead use evidence-based medicine. This would help avoid early medication resistance while also conserving resources and relieving demand on ACT^50^.

A high degree of adherence to the results of the malaria test complemented the high rate of test-before-treatment practiced in the private institutions. The perception of RDT's value for diagnosing and treating malaria among health professionals in private facilities was favourable. The positive acceptance of RDT and compliance with the test result may have been influenced by health professionals' perception that RDT took a “short time”^24^. With research focused on addressing other areas of concern, these encouraging findings offer a significant possibility for the expansion of RDT use in formal private health facilities. The high testing rate in private healthcare institutions may also be due to financial incentives, concern about legal action, or simple adherence to regulations regarding policy and practice. In private health facilities, the current state of testing for malaria before treatment was excellent. Compliance with the result of the RDT will go a long way in reducing presumptive treatment of every febrile illness as malaria and will help to address emerging resistance to Artemisinin-based combination treatment (ACT).

## Conclusion

From the review, there was poor uptake of malaria preventive strategies generally. Eight out of the twelve selected articles (66.7%) reported poor uptake while only four articles (33.3%) showed good uptake of malaria control preventive strategies. In the area of use of LLIN, two out of the five publications^18^,^19^, reported good uptake while three reported poor uptakes. The only two articles selected in the area of mRDT^23^, ^24^ reported good uptake of mRDT and adherence to malaria test results. The least was the uptake of IPTp-SP where all five publications reviewed reported poor uptake. These findings have revealed why the mortality and morbidity from malaria infection is still very high in Nigeria.

The poorly organized and underfunded health care system in Nigeria was among the reasons responsible for the low uptake or higher rates of missed opportunities for IPTp-SP that were seen in the research. Ignorance, cultural, and socioeconomic limitations are other causes. The government must work harder to improve its policy frameworks and programmatic initiatives to address these concerns in light of these results. The emphasis should be on implementation of the prevention strategies, stakeholders and community engagement to address the socio-cultural barriers identified in the review. Health education and implementable policy on health care strengthening will also help in the uptake of the prevention strategies.

## Study limitation

The study was constrained by the use of solely PubMed for data extraction. Although PubMed is regarded as one of the best and most widely used databases for health sciences papers that is simple to access, it's possible that extra publications were missed since other databases couldn't be searched. The number of publications used for RDT was small.
